# Methods to control disclosure risk of synthetic data created by National Statistics Agencies

**DOI:** 10.23889/ijpds.v8i2.2301

**Published:** 2023-09-14

**Authors:** Gillian Raab

**Affiliations:** 1 University of Edinburgh, Edinburgh, United Kingdom

## Objectives

With the recent explosion of interest in using synthetic data (SD) for disclosure control many NSAs are releasing, or considering releasing. synthetic versions of their administrative data. This presentation will review the methods that NSAs can use to limit the disclosure risk of any planned release of synthetic data.

## Methods

This paper will review the ways in which methods of creating can be adapted to control the disclosure risk that could arise by the release of such data either to trusted researchers or to a wider group. Methods that will be evaluated will include:

1. The use of Statistical Disclosure Control (SDC) methods on the synthetic data before its release
2. Selecting methods producing low fidelity synthetic data
3. Adapting the synthesis method until it satisfies measures of disclosure risk
4. Incoporating differential privacy (DP) into the method of creating synthetic data

## Results

NSAs can use different methods to create SD based on real data (RD); see e.g. https://unece.org/info/publications/pub/373531. Tthe disclosure risk of SD depends on the context of its release, to whom, in what environment etc. Even if the planned method of release ensures low disclosure risk, NSAs will want to know what the disclosure risk might be if the SD got into the wrong hands.

The SD can reveal that an identified person is in the RD (identity disclosure) or can disclose information about other measures for an individual that are part of the RD. Measures of identity disclosure and attribute disclosure are described. Results will be presented on the disclosure risk of examples of SD created for real examples by the methods 1 to 4.

## Conclusion

Each of the methods 1 to 4 have strengths and weaknesses. Methods 2 and 4 will be ruled out for many applications because of poor fidelity to the RD. A practical way forward is suggested by combining methods 1 and 3.

